# Comparative Effects of E-Cigarette Aerosol on Periodontium of Periodontitis Patients

**DOI:** 10.3389/froh.2021.729144

**Published:** 2021-09-07

**Authors:** Fangxi Xu, Eman Aboseria, Malvin N. Janal, Smruti Pushalkar, Maria V. Bederoff, Rebeca Vasconcelos, Sakshi Sapru, Bidisha Paul, Erica Queiroz, Shreya Makwana, Julia Solarewicz, Yuqi Guo, Deanna Aguallo, Claudia Gomez, Donna Shelly, Yindalon Aphinyanaphongs, Terry Gordon, Patricia M. Corby, Angela R. Kamer, Xin Li, Deepak Saxena

**Affiliations:** ^1^Department of Molecular Pathobiology, New York University College of Dentistry, New York, NY, United States; ^2^Department of Epidemiology and Health Promotion, New York University College of Dentistry, New York, NY, United States; ^3^Department of Medicine, New York University School of Medicine, New York, NY, United States; ^4^Department of Population Health, New York University School of Medicine, New York, NY, United States; ^5^Department of Environmental Medicine, New York University School of Medicine, New York, NY, United States; ^6^Department of Oral Medicine, School of Dental Medicine, University of Pennsylvania, Philadelphia, PA, United States; ^7^Department of Periodontology and Implant Dentistry, New York University College of Dentistry, New York, NY, United States

**Keywords:** e-cigarettes, aerosol, smoking, periodontal disease, host response, oral health, longitudinal study

## Abstract

**Introduction:** Tobacco use is one of the main causes of periodontitis. E-cigarette are gaining in popularity, and studies are needed to better understand the impact of e-cigarettes on oral health.

**Objective:** To perform a longitudinal study to evaluate the adverse effects of e-cigarettes on periodontal health.

**Methods:** Naïve E-cigarette users, cigarette smokers, and non-smokers were recruited using newspaper and social media. Age, gender, and ethnicity, were recorded. Participants were scheduled for two visits 6 months apart. At each visit, we collected data on the frequency and magnitude of e-cigarette and cigarette use, and alcohol consumption. Carbon monoxide (CO) levels, cotinine levels, salivary flow rate, periodontal probing depth (PD), bleeding on probing (BoP), and clinical attachment loss (CAL) were also determined at both baseline and follow-up visits and compared between groups with two-way repeated measures ANOVA. Periodontal diagnosis and other categorical variables were compared between groups with the chi-square statistic and logistic regression.

**Results:** We screened 159 subjects and recruited 119 subjects. One-hundred-one subjects (31 cigarette smokers, 32 e-cigarette smokers, and 38 non-smokers) completed every assessment in both visits. The retention and compliance rate of subjects was 84.9%. The use of social media and craigslist was significant in recruiting e-cigarette subjects. Ethnicity and race differed between groups, as did average age in the male subjects. Carbon monoxide and salivary cotinine levels were highest among cigarette smokers. Bleeding on probing and average PDs similarly increased over time in all three groups, but CAL uniquely increased in e-cigarette smokers. Rates of severe periodontal disease were higher in cigarette smokers and e-cigarette users than non-smokers, but interpretation is confounded by the older age of the cigarette smokers.

**Conclusion:** Among the recruited participants, CAL after 6 months was significantly worse only in the e-cigarette smokers. This study design and protocol will assist in future larger studies on e-cigarette and oral health.

## Introduction

Periodontitis, also known as gum disease, is a chronic, polymicrobial inflammatory disease affecting the tissue supporting the tooth. One of the main risks for periodontitis is smoking, as it alters the microbiome [1] of the oral cavity and the host immune response [2], causing the oral tissue to become vulnerable and susceptible to disease. Previous studies have demonstrated that the use of tobacco-containing products could potentially lead to oral manifestations, such as mucosal lesions (e.g., leukoplakia, candidiasis, nicotine stomatitis), plaque formation, teeth staining, gingivitis, periodontitis, tooth loss, failure of prosthetic and surgical treatments, and increased risk of oral cancer [3–5]. Over the years, conventional cigarette smoking has declined; however, the use of emerging tobacco products, such as electronic cigarettes (e-cigarettes) has increased [6]. E-cigarette are non-combustible battery-operated devices that allow users to inhale an aerosol mixture that typically contains propylene glycol and/or glycerin with or without nicotine and other additives [7]. The performance of e-cigarettes varies among different brands [8], and the manufacture and components of E-cigarette product are regulated by FDA. It has been proposed that e-cigarettes serve as a strategy of smoking cessation or a less harmful replacement for conventional cigarette [9, 10]. However, the data is inconclusive [11]. Switching from smoking to e-cigarettes reduces the number of cigarettes smoked; however, it does not result in complete withdrawal, and the risk of developing smoking-related diseases, particularly oral diseases, remains a high possibility [12, 13]. Moreover, the CDC recently reported 2,668 hospitalized e-cigarettes use-associated lung injury cases or deaths [14]. Among those cases, 15% of patients were under 18 years old, and 37% of patients were 18–24 years old [14]. These pathologies suggest that e-cigarettes can significantly damage various tissues, including oral tissues. As the popularity of e-cigarettes use increases, and the potential for damage exists, it is necessary to investigate the impact of e-cigarette use on oral health.

The E-cigarette aerosol includes, but is not limited to, tobacco-specific nitrosamines, aldehydes, metals, and volatile organic compounds [15]. These compounds could potentially alter the oral microbiome and have adverse effects on oral health. Disturbance of the oral microbiome, particularly commensal microorganisms, might lead to dysbiosis and increase pathobionts, which might lead to oral diseases, such as periodontal disease. Dysbiosis might, in turn, activate different inflammatory pathways and, subsequently, lead to systemic health conditions, such as respiratory [16, 17], immune [18], and cardiovascular complications [19]. Furthermore, our recent study showed that e-cigarette aerosol exposure caused elevated concentrations of proinflammatory cytokines (IL)-6 and IL-1β, thus potentially increasing susceptibility to periodontal disease [20].

Clinical parameters of periodontal inflammation include clinical attachment loss (CAL), increased probing depth (PD), and bleeding on probing (BoP) [21, 22]. Studies have shown that clinical parameters of periodontitis are poorer in cigarette smokers compared to non-smokers [23]. Few self-reported studies have shown that people using e-cigarettes have bad periodontal health [24–26]. Most of the studies are cross-sectional and they did not provide enough information how e-cigarette aerosol alters periodontal health during the course of time. Here, we designed a longitudinal study to present a demographic description of our population and compare e-cigarette users with cigarette smokers and non-smokers. The primary hypothesis is that clinical parameters of periodontal disease are worse in cigarette smokers and e-cigarette users compared to non-smokers. The findings of this study will help to understand the potential risks associated with e-cigarette use.

## Methods

### Ethical Guidelines

The approval of the study protocol, informed consent form(s), and all subject materials were obtained by the Institutional Review Board (IRB) of the New York University Langone Medical Center. Before any study-related assessment, the participants received a detailed explanation of the research study and procedures. The informed consent of each participant was obtained prior to sample collection, and a copy of the consent form was provided to each participant for their record. Information regarding the risks and possible benefits of study participation was provided, and participants were informed that they might withdraw consent at any time throughout the course of the study. All STROBE guidelines were followed.

### Study Design and Participants

**Figure 1:**
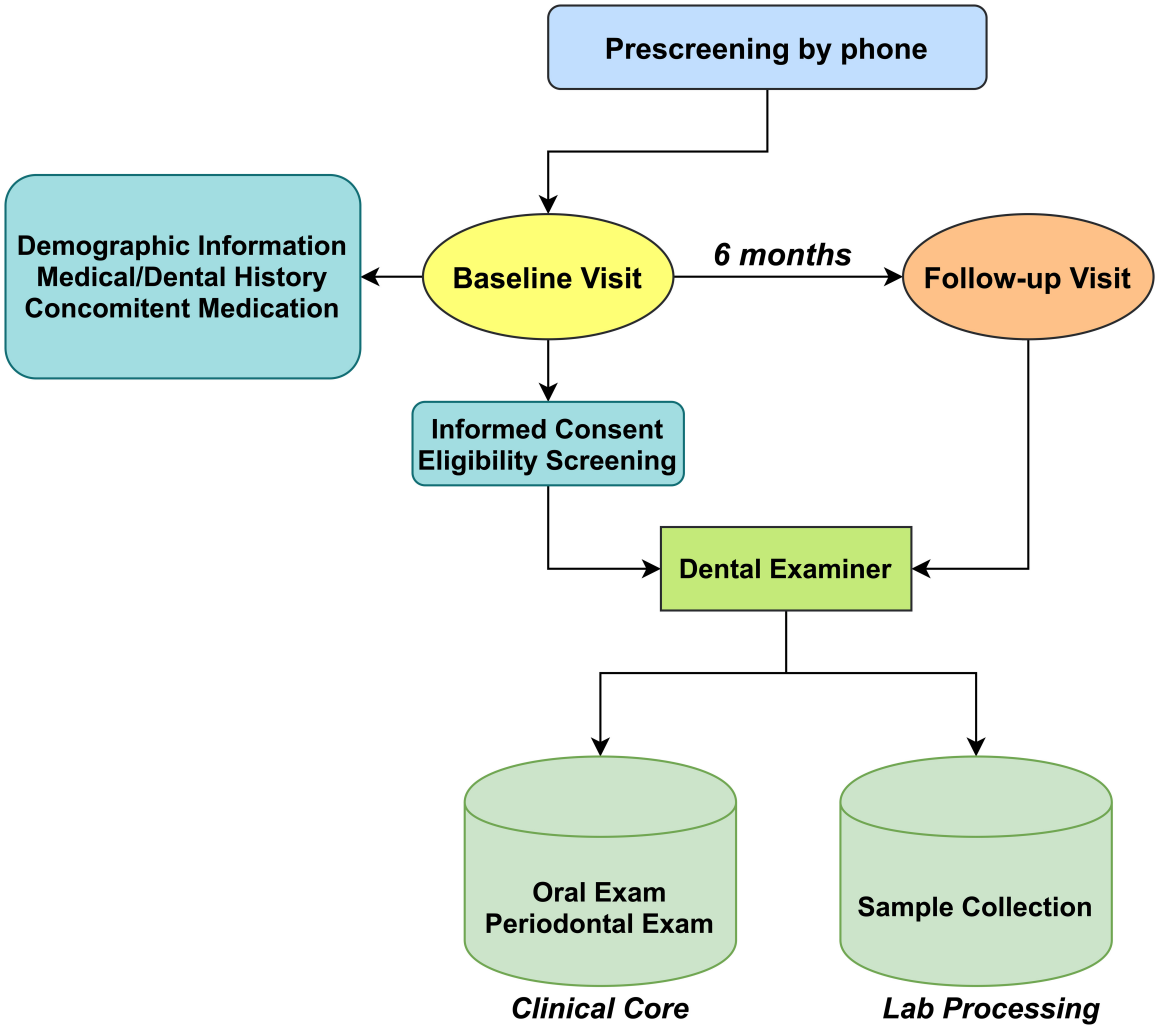
Schematic showing the flow of subject recruitment and sample collection. The types of samples collected and their transit from collection points to a processing laboratory where they were labeled and entered into a database.

The present study aimed to compare clinical indicators (PD, BoP, CAL) of periodontitis among cigarette smokers, e-cigarette users, and non-smokers. We planned to recruit 120 subjects with 40 in each group (cigarette smokers, e-cigarette users, and non-smokers) which will be sufficient to detect a group difference of one standard deviation in a two-tailed independent samples *t*-test with a power of 99%. Figure 1 shows the workflow of the study. Study visits were conducted at the NYUCD Bluestone Center for Clinical Study. Upon obtaining informed consent and completion of a standardized oral health questionnaire, further social, medical, and dental history, and concomitant medication use were recorded, and we confirmed that none of the recruited subjects was using anti-inflammatory drugs. Subjects were asked to report on the frequency and intensity of tobacco and alcohol use as they have been considered as important risk factors for periodontal disease [27, 28], Previous and current health conditions, surgeries, medications, and symptoms of existing conditions were also recorded. Carbon monoxide (CO) levels were assessed, and saliva was collected for the determination of cotinine levels. Periodontal examinations were performed by a calibrated examiner, and subgingival plaque and saliva samples were collected for microbiome analysis (reported elsewhere) [20]. A follow-up visit (V2) was scheduled 6 months [28–30] after the baseline visit (V1), and the protocol was repeated along with the assessment of adverse events. Participant charts were assigned an identification number and secured at the NYU's Bluestone Center for Clinical Research.

To be eligible for the study, participants were required to meet conditions specific to each group. A cigarette smoker was defined as someone who, at the time of the study, smoked at least 10 cigarettes daily for a period of 12 months or more. E-cigarette users were defined as a non-cigarette smoker who used minimum of 0.5–1 e-cigarette daily for minimum of the last 6 months. Lastly, a non-smoker was defined as someone who never smoked a cigarette or used an e-cigarette in their lifetime.

### Participants Enrollment, Recruitment, and Eligibility

Recruitment of participants was managed by the study coordinator and personnel from New York University's Bluestone Center for Clinical Research, New York, NY. Study flyers were displayed at NYUCD Television screens, NYU primary care, and dental clinics, as well as the Health and Hospital Corporation's primary care sites. Additionally, the study advertisement was posted in local newspapers on Craig's list and Facebook, which has been an effective tool for recruitment.

Participants were required to be 21 years of age, to have a minimum of 16 teeth, including eight posterior teeth, and diagnosed with mild, moderate, or severe periodontal disease [21, 22]. The exclusion criteria were as follows: (a) a medical condition (including uncontrolled diabetes and HIV); (b) recent febrile illness that delays or precludes participation; (c) pregnancy or lactation; (d) history of radiation therapy to the head and neck region; (e) antibiotic use or professional dental cleaning within 1 month; (f) enrollment in other studies; (g) or presence of oral mucosal lesions, such as leukoplakia, herpes labialis, and candidiasis. In addition, non-smoker subjects were excluded from the study if the CO level was at least seven parts per million (ppm), calling into doubt their non-smoking (NS) status.

Among the 159 subjects who attended the screening visits, a total of 119 subjects participated in our study; 39 non-smokers, 40 exclusively conventional cigarette smokers, and 40 exclusively e-cigarette users successfully enrolled and completed all the assessments of baseline visits. Of these participants, 101 (38 non-smokers, 31 cigarette smokers, and 32 e-cigarette users) have completed the follow-up examination 6 months after the baseline visit (Figure 1). Participants who did not complete the follow-up visit were either lost to follow-up, withdrew for personal reasons (such as relocation).

### Questionnaire

A questionnaire was developed from the Center for Disease Control and Prevention (CDC) oral health questionnaire [31] and completed at baseline and follow-up visits. The questionnaire included eight questions related to periodontal health and past treatment, tooth status, and how many times they used floss and mouthwash during the preceding 7 days.

### Clinical Data Collection

Subject's sex, age, ethnicity, race, nicotine (conventional cigarette and e-cigarette [daily puffs]), and alcohol use history were recorded. Subjects who were eligible and included in this study were asked to follow up daily via specially created text messaging applications to monitor smoking and e-cigarette status for compliance. The information collected was secured by the REDCap database and Twilio software. The subject's identification was encrypted, and the information was transferred to the NYUCD database. The database includes the medical history, dental history, and periodontal status of the subjects.

### Assessment of CO Levels

To confirm the smoking status of each participant, carbon monoxide (CO) levels were tested by CO Smokerlyzer (Smokerlyzer, Covita, Santa Barbara, CA) according to the manufacturer's instructions. Participants were instructed to inhale deeply and hold their breath for fifteen seconds before slowly exhaling into the device. Based on the CO test results, participants were categorized into one of five groups: NS (0–6 ppm), low addicted smokers (LAS) (10–15 ppm), moderately addicted smokers (MAS) (16–25 ppm), heavily addicted smokers (HAS) [27–36], and very heavily addicted smokers (VHAS) (≥36 ppm) [20].

### Oral Examination

Oral examination was performed by three different calibrated periodontists or dental hygienists. Oral examination was completed at each visit and included: mucosal assessment of lower and upper lip, hard and soft palate, uvula, the floor of the mouth, tongue, tonsils, and labial and buccal mucosa. If any abnormality (such as candidiasis, herpes labialis, aphthous stomatitis) was present, the participant was referred to an oral medicine specialist.

### Gingival and Periodontal Assessment

A full mouth examination was performed to assess the periodontal condition. Periodontal measurements were recorded at six sites per tooth (mesio-buccal, buccal, disto-buccal, mesio-lingual, lingual, and disto-lingual) on all teeth present and included the following: [1] probing depth (PD) defined as the distance from the free gingival margin to the depth of the pocket; [2] distance from the free gingival margin to the cement enamel junction (CEJ); and [3] presence or absence of BOP. Clinical attachment loss was then calculated by subtracting the CEJ measurement from the PD. For analysis, the percentage of bleeding sites was determined by dividing the number of sites that bled by the total number of sites sampled and multiplying by 100. Probing depth and CAL were summarized as the average PD and CAL among the sampled sites.

The classification of mild, moderate, or severe periodontal disease followed the definition given by the CDC in collaboration with the American Academy of Periodontology (CDC-AAP) [32]. Mild periodontitis was defined as ≥ two interproximal sites with ≥3 mm attachment loss, and ≥2 mm interproximal sites with PD ≥ 4 mm (not on the same tooth), or one interproximal site with PD ≥ 5 mm. Moderate periodontitis was defined as two or more interproximal sites with ≥4 mm clinical AL (not on the same tooth), or two or more interproximal sites with PD ≥ 5 mm, also not on the same tooth. Severe periodontitis was defined as having two or more interproximal sites with ≥6 mm AL (not on the same tooth), and one or more interproximal site(s) with ≥5 mm PD [20].

### Saliva Sample Collection and Flow Rate Assessment

Participants were asked to chew paraffin wax pellets (Gleegum, Verve Inc., Providence, RI) to stimulate salivary secretion. After chewing gum for 30 s to 1-min, participants were asked to expectorate 10 ml saliva into a sterile graduated 50 ml centrifuge tube on ice. The amount of saliva was measured after 5 min. If the measured amount was <5 ml, participants were asked to keep expectorating. The salivary flow rate was calculated based on recorded data at 5 min. Saliva samples were stored on ice and delivered to the clinical site's laboratory for processing. Some saliva (1 mL) was utilized immediately for the cotinine level evaluation (Nic Alert kit, Salimetrics, State College, PA). Retained samples were aliquoted, preserved with phenylmethylsulfonyl fluoride (PMFS), and subjected to aprotinin immune mediator analysis. Aliquots were also saved for microbiome analysis. All the samples were stored at −80°C.

### Statistical Analysis

All data were exported from NYULMC REDCap, and statistical analysis was performed using IBM SPSS (v26, IBM Corp., Armonk, NY). Analysis of continuous measures (a measure of CO, salivary flow rate, PD, BOP, and CAL) compared means from the three groups over time using a two-way mixed model analysis of variance (Repeated measures procedure) followed by Tukey's honestly significant difference (HSD) test. If confronted with heterogeneous variances, the Kruskal-Wallis or Welch test was substituted. Partial eta-squared (*p*η^2)^ is shown as the measure of effect size for significant effects. Some analyses evaluated confounding due to group differences in age, race and sex when those variables were correlated with the outcome measure. Differences between groups in rates of periodontal diagnosis were evaluated using the chi-square test, and changes in diagnosis over time within groups were evaluated using the McNemar test. Logistic regression was then used to evaluate confounding between-group differences potentially attributable to demographic variables. It was estimated that a sample size of 40 per group was sufficient to detect a group difference of one standard deviation in a two-tailed independent samples *t*-test with a power of 99%. In the case of PD, this means that the planned enrollment would provide power of 99% to detect a minimal difference between group means of 0.5 mm (based on the average SD in the current data). With attrition, obtained power between the two smallest samples was reduced to about 97%. For within group comparisons, the planned sample would also provide power of 99% to detect a 0.7 SD change in means over time in a paired samples *t*-test, or about a 0.35 mm change in PD. With attrition, power was reduced to about 97%. Thus, the study was adequately powered to detect clinically significant differences in PD both between and within groups. *P*-values < 0.05 were considered statistically significant.

## Results

### Participant's Demographics

A total of 101 subjects completed the baseline and 6-month follow-up evaluations and sample collections: 31 were cigarette smokers, 32 were e-cigarette smokers, and 38 were non-smokers. The demographic characteristics of the study subjects are shown in Table 1. Seventy percent of the subjects were male. Among males, non-smokers were significantly younger than e-cigarette smokers, and e-cigarette smokers were significantly younger than cigarette smokers. Most non-smokers were Asian, most cigarette smokers were Black, and most e-cigarette smokers were White.

**Table 1:** Demographics.

	**Cigarette smokers**	**E-cigarette smokers**	**Non-smokers**	***p*-Values**
***N***	31	32	38	
**Sex (% male)**	77.4	78.1	57.9	0.11
**Age (year), M (SD)**				
Male	46.9 (10.1)^b^	36.0 (9.8)^c^	28.8 (6.1)^a^	<0.001^*^
Female	46.6 (11.5)	38.7 (10.6)	39.0 (14.1)	0.40
**Ethnicity (% Hispanic)**	6.5	22.6	23.7	0.13
**Race (%)**				0.002
White	32.3	56.3	26.3	
Black	54.8	34.4	28.9	
Asian	9.7	6.3	42.1	
Other	3.2	3.1	2.6	

* *One-way analysis of variance and Tukey post-hoc test*.

a;b;c *Unlike superscripts indicate significantly different group means(p < 0.05)*.

### Tobacco and Alcohol Use

By design, each group was enrolled considering the inclusion criteria for the smoking behavior. Table 2A shows that e-cigarette smokers consumed an average of <1 cartridge per day at each study visit. However, the average puffs per day of e-cigarettes declined between the baseline visit and follow-up visit (*p* = 0.03). By contrast, cigarette smokers maintained a constant average use over time, of about 13 cigarettes per day (*p* = 0.70).

**Table 2A:** Smoking behavior.

	**Baseline visit**	**Follow-up visit**	***p*-Values**
**E-cigarette Smoker**			
E-cigarettes/day	0.78 (0.25)	0.74 (0.51)	0.70
Puffs/use	151.3 (104.4)	94.4 (99.1)	0.03
**Cigarette Smoker**			
Cigarettes/day	13.5 (4.8)	12.3 (4.4)	0.17

Table 2B shows that approximately half of the subjects in each study group reported using alcohol on both visits. Each group reported drinking about two times per week, consuming two or three drinks each time. Although not reaching statistical significance, e-cigarette smokers tended to drink more often than others (*p* = 0.1).

**Table 2B:** Alcohol consumption.

	**Alcohol User at f/u, *n* (%)**	Drinking (days/week), Mean (SD)^*^	# Drinks/drinking day, Mean (SD)^*^
Cigarette smokers	14 (48.4)	2.0 (1.2)	2.8 (2.3)
E-cigarette smokers	15 (53.1)	2.4 (1.2)	4.1 (2.3)
Non-smokers	18 (50.0)	1.5 (1.2)	2.6 (2.3)
*p*-Values	0.93	0.10	0.16

* *The analysis of alcohol use (days/week and # drinks/day) is based on the consuming participants*.

### The CO, Cotinine Level, and Saliva Flow Rate Across Groups

**Figure 2:**
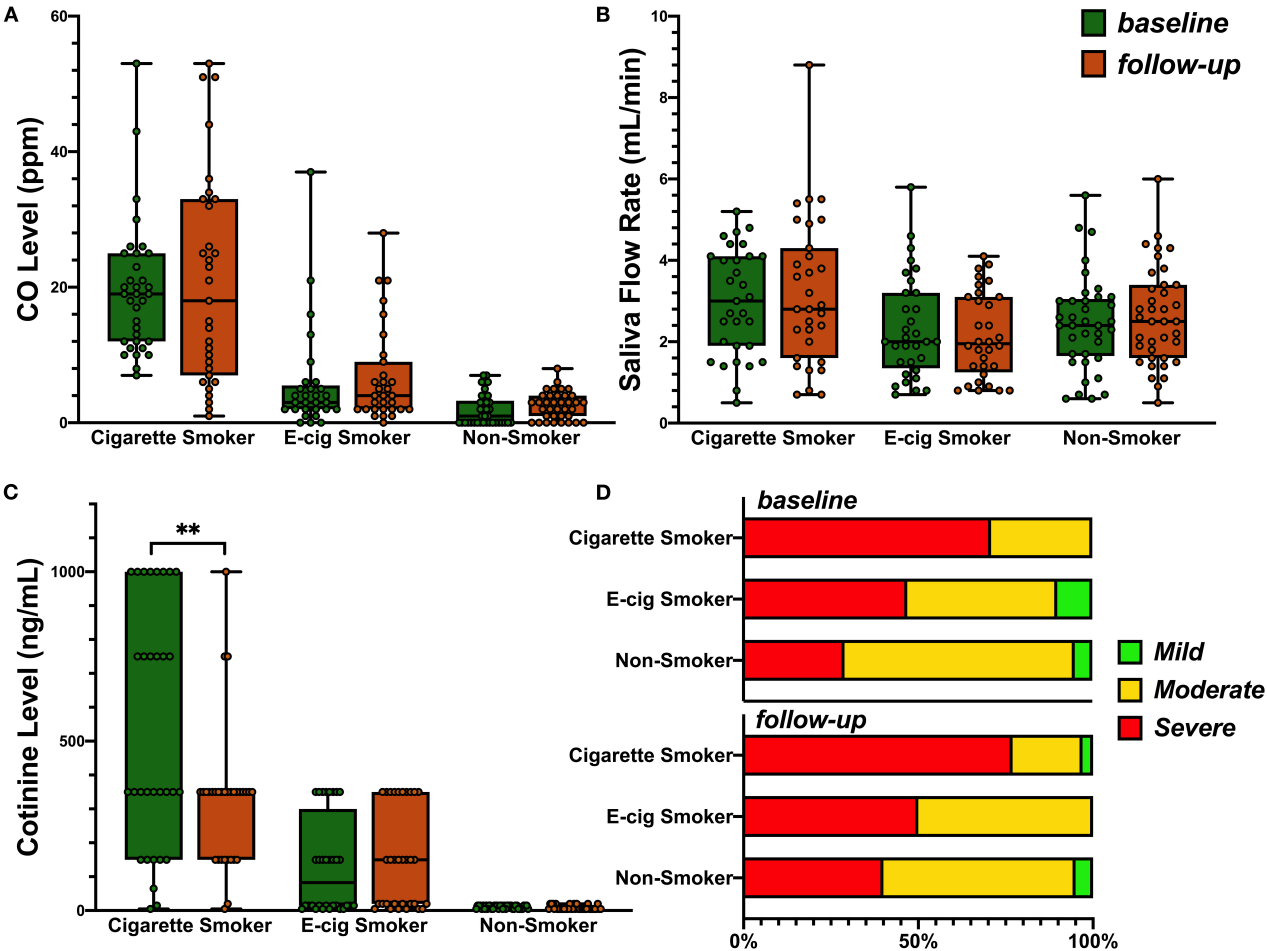
**(A)** Levels of breath carbon monoxide (ppm) across the subjects in the cigarette smokers, e-cigarette smokers, and the non-smokers at base line and 6-month follow-up: 0–6 ppm, Borderline (BdL): 7–9 ppm, low addicted smoker (LAS): 10–15 ppm, moderate addicted smoker (MAS): 16–25 ppm, heavily addicted smoker (HAS): 26–35 ppm, and very heavily addicted smoker (VHAS): 36+ ppm. Group mean was used for ANOVA (*p* < 0.001) as no interaction of time and group was observed. **(B)** Saliva flow rate in three groups at base line and follow-up. Group mean was used for ANOVA (*p* = 0.02) as no interaction of time and group was observed. **(C)** Distribution of salivary cotinine levels in the participants of the three groups. Kruskal-Wallis test was used and *p* < 0.001 for both visits. Paired *t*-test (*p* = 0.002) was then used to evaluate the change over time in each group. *P* value less than 0.01 was summarized with 2 asterisks. **(D)** Prevalence of periodontal disease in all three groups from baseline to follow-up visit.

Cigarette smoking subjects showed higher mean levels of carbon monoxide than e-cigarette smokers or non-smokers (Figure 2A, 20.8 vs. 5.8 and 2.8 ppm, respectively, *p* < 0.001), as well as higher mean levels of salivary flow rate (Figure 2B, 3.1 vs. 2.2 and 2.5 ml/min, respectively, *p* = 0.02). Consistent with their smoking behaviors, salivary cotinine levels were higher in cigarette smokers than e-cigarette smokers, who were higher than non-smokers at each test period (Figure 2C Kruskal-Wallis test, all *p* < 0.001). Figure 2C also shows a reduction in cotinine levels over time in the cigarette smokers (paired sample *t*-test, *p* = 0.002). Analysis failed to show an interaction between group and time on any of these measures.

### Gingival Health

#### Bleeding on Probing

The mean percentage of BoP in the three groups is shown in Table 3A. Analysis showed similar levels at baseline (*p* = 0.37) and an increase over time from 56% at baseline to 64% at follow-up (*p*η^2^ = 0.05, *p* = 0.03) and statistically similar changes over time in the three groups (*p* = 0.30). Neither sex, race, nor age were related to BOP, and were not added to the model in order to evaluate confounding.

**Table 3A:** Rates of BoP and periodontal disease severity, and levels of, probing depth, and clinical attachment loss as a function of group and time.

	**Visit**	**Non-smokers**	**E-cigarette smokers**	**Cigarette smokers**	***p*-Values**
Bleeding on Probing (%), Mean (SD)^*^	Baseline	52.6(32.1)	53.0 (32.5)	61.5 (27.6)	0.03
	Follow-up	68.3(24.4)	57.0 (32.6)	66.2 (30.5)	
Probing depth (mm), Mean (SD)	Baseline	2.7 (0.4)^a^	3.0 (0.6)^a,b^	3.1 (0.7)^b^	0.001^*^
	Follow-up	2.9 (0.4)^a^	3.1 (0.7)^a,b^	3.2 (0.6)^b^	0.01^*^
Clinical attachment loss (mm), Mean (SD)	Baseline	2.2 (0.9)^a^	2.8 (1.5)^b^	3.5 (1.1)^b^	<0.001^*^
	Follow-up	2.2 (0.7)^a^	3.1 (1.4)^b^	3.4 (1.1)^b^	<0.001^*^
Periodontal disease (% progressed) +	Follow-up	18	29	44	NA

* *Welch test for heterogeneous variances and Tukey post-hoc test*.

ab *Unlike superscripts indicate significantly different group means(p < 0.05)*.

+ *Cases with mild and moderate periodontal disease used in this analysis*.

#### Probing Depth

Table 3A shows mean (SD) PD for each group at both evaluations. Analysis showed that PD varied by group (*p*η^2^ = 0.05, *p* = 0.02). Probing depth was significantly greater in the cigarette smoker group [M (SD) = 3.0 (0.65) mm] than the non-smoker group [M (SD) = 2.8 (0.4) mm]; the e-cigarette smoker group, however, was statistically similar to both other groups [M (SD) = 3.1 (0.65) mm]. Over time, there was an increase in mean (SD) PD from 2.9 (0.60) to 3.1 (0.59) mm (*p*η^2^ = 0.20, *p* < 0.001), a change that was comparable in the three groups (*p* = 0.97). While PD was related to race (highest in Black subjects), adjusting these PD analyses for race failed to change conclusions.

#### Clinical Attachment Loss

Table 3A shows mean (SD) CAL for each group at both evaluations. Analysis showed that CAL varied by group [*p*η^2^ = 0.20, *p* < 0.001]. It was greater in the e-cigarette smokers [M (SD) = 2.9 (1.5) mm] and cigarette smokers [M (SD) = 3.5 (1.1) mm] than in the non-smokers [M (SD) = 2.2 (0.8) mm] (*p* < 0.001). While there was no general increase in CAL with time, the increase of about 2 mm in CAL in the e-cigarette smokers was greater than that seen in the non-smokers or the cigarette smokers, who did not change (Table 3A, interaction *p*η^2^ = 0.10, *p* < 0.001). CAL was also related to race (highest in Black subjects), but adjusting these CAL analyses for race failed to change conclusions.

#### Periodontal Diagnosis

Figure 2D shows that the prevalence of severe periodontal disease was higher among cigarette smokers than the other groups, at both visits (all *p* < 0.05), while rates of severe disease were comparable in non-smokers and e-cigarette smokers. As the prevalence of severe periodontal disease also increased with age (baseline *r* = 0.43; follow-up *r* = 0.47, both *p* < 0.001), we regressed group and age on the rate of severe periodontal disease. Table 3B shows that group differences were indeed confounded with age. This leaves one without a definitive conclusion regarding the effect of cigarette smoking vs. age on the prevalence of severe disease. Sex and race were not related to diagnosis. The last row of Table 3A shows that over time, fewer non-smokers progressed to more severe disease than e-cigarette smokers, who progressed less than cigarette smokers. Nevertheless, analysis failed to show that the prevalence of severe periodontal disease changed from baseline to follow-up visits in any group (McNemar test: Cigarette smoker, *p* = 0.69; e-cigarette smoker, *p* = 1.00; Non-smoker, *p* = 0.22).

**Table 3B:** Logistic regression analysis relating rates of severe periodontal disease to cigarette smoking status and age.

**Baseline visit**				**Follow-up visit**			
Step1^*^		**OR (CI)**	***p*-Values**	Step1^*^		**OR (CI)**	***p*-Values**
	Cigarette smoking	4.1 (1.7–10.3)	0.002		Cigarette smoking	4.3 (1.6–11.3)	0.003
Step2^*^				Step2^*^			
	Cigarette smoking	2.0 (0.7–5.7)	0.18		Cigarette smoking	1.8 (0.6–5.4)	0.31
	Age (years)	1.07 (1.03–1.12)	0.002		Age (years)	1.09 (1.04–1.15)	<0.001

* *Smoking status was entered into the regression on step 1, and age was then added in step 2. When adjusting age, smoking status no longer predicts severe periodontal disease*.

To summarize, these data suggest that cigarette smokers started the study with the worst periodontal health. E-cigarette smokers presented with intermediate levels of disease, and non-smokers were in the best health at baseline. All subjects experienced more BOP and increased PD over time, and e-cigarette smokers had an increased risk of CAL progression. Overall periodontal diagnoses did not change.

## Discussion

A great deal of literature is available on conventional tobacco products, but limited data is available on the effects of e-cigarette on oral health. To address this, we conducted a clinical study to compare e-cigarette users with cigarette smokers and non-smokers to determine the impact of e-cigarette use on oral health, particularly periodontal health. The initial interaction of e-cigarette aerosol mixtures occurs largely in the oral cavity, where nicotine and other compounds are expected to be most active, and the exposure is most intense. A recent online survey of 543 e-cigarette users indicated that most negative health effects were observed in the mouth and throat [33].

The cigarettes smoking behavior did not change in the use of cigarette or e-cigarette per day but there was a significant change in the number of puffs per e-cigarette (151.3 puffs at baseline and 94.4 puffs at 6 month follow up), suggesting more intensive puffing and higher consumption of nicotine per puff as they get adapted to e-cigarette. Studies have shown that puffing patterns associated with nicotine strength or e-liquids and voltage used in e-cigarette which result in higher toxicant exposure [34–36].

The prevalence of severe periodontal disease was higher than expected in all groups (Figure 2D). This is likely because we only recruited those who had at least mild levels of periodontal disease. Bleeding on probing and PD increased over time to similar extent in all subjects, but CAL increased only in the e-cigarette group. Overall, the percentage of severe periodontal disease was much higher in the e-cigarette group than among the non-smokers. Other studies have reported similar findings that e-cigarette users had higher chances of developing periodontal disease [24, 25]. We reported previously that there was a microbiome shift in e-cigarette users' oral cavity and an increase of the levels of periodontal inflammatory indicators making them more likely to get periodontal infection compared to non-smokers [20]. At the same time, the differences observed between the e-cigarette users and cigarette smokers maybe attributed to the carcinogenic and toxic compound present in the smoke of the conventional cigarettes as compared to e-cigarettes [37].

A limitation of the study design was that the groups were not matched for age; although it is difficult as e-cigarette users are much younger than the cigarette smokers but this should be a considered in all future e-cigarette clinical research. E-cigarette users were much younger than the cigarette smokers, and that cofounded some of our findings [22, 38–43]. Age was also considered a confounder of the relationship between cigarette smoking status and the rate of severe periodontitis. As such, age appears to be the more parsimonious explanation of higher rates in those participants. Another variable that could have led to differential outcomes is the race, which was not evenly distributed in the study groups. We notice that most non-smokers were Asian, most cigarette smokers were black, and most e-cigarette smokers were white, suggesting disparity among smokers and e- cigarette users. It has been reported by the CDC that US Blacks and Hispanics show poorer oral health compared to Whites and Asians [44]. Nevertheless, while the majority of the subjects in the non-smoker group were Asian, e-cigarette users were primarily White, and cigarette smokers were primarily Black, the analysis showed effects of race on periodontal status. Thus, race should also be controlled in future work. Finally, the paucity of changes over time may be the result of a too short follow-up interval. Future work should consider longer intervals.

Modified questionnaires, with more precise information on the subject's social practices, including alcohol usage and their dental hygiene routine, will control for confounding factors in the study. The study design could be further improved if social and education status were evaluated to check whether there is a relationship between higher education, better oral health, and e-cigarette use. Different types and brands of e-cigarette also need to be considered as the variation in e-liquid components, flavoring agents, and voltages may contribute to the nicotine yield in the e-cigarette aerosol [45].

To our knowledge, this is the first clinical research report on the oral health impacts of vaping (e-cigarette use) relative to cigarette smokers and non-smokers. The described study design and its limitations can guide future larger studies on e-cigarette use.

## Data Availability Statement

The raw data supporting the conclusions of this article will be made available by the authors, without undue reservation.

## Ethics Statement

The studies involving human participants were reviewed and approved by Institutional Review Board (IRB) of the New York University Langone Medical. The patients/participants provided their written informed consent to participate in this study.

## Author's Note

The results of this study can be used by scientist and clinicians when designing clinical research which they may use to study periodontal disease.

## Author Contributions

FX and EA: carried out REDCap data entry, data analyses and interpretation, and manuscript preparation. SP and BP: carried out sample collection, data analyses and interpretation, and manuscript preparation. MB, SS, SM, JS, and YQ: carried out clinical data analysis, data entry, technical lab work and statistical analyses, and manuscript preparation. MJ: took part in the study design, statistical analyses, and critical review. EQ and RV: carried out subject recruitment. DA performed oral exam and clinical sample collection. CG performed oral exam and clinical sample collection. AK performed oral exam, clinical sample collection, and analyses. DSh performed subject recruitment. YA managed REDCap, clinical data and electronic messaging system. TG assisted in aerosol generating machine and manuscript preparation. PC provided assistance in subject recruitment and clinical sample collection. XL and DSa: conceived, designed, supervised, analyzed, interpreted the study, provided critical review, and manuscript preparation. All authors contributed to the article and approved the submitted version.

## Funding

This research project was supported by NIH grants DE025992 (DSa and XL), DE027074 (DSa and XL), CA206105 (DSa), and the NYU Mega grant initiative (DSa and XL).

## Conflict of Interest

The authors declare that the research was conducted in the absence of any commercial or financial relationships that could be construed as a potential conflict of interest.

## Publisher's Note

All claims expressed in this article are solely those of the authors and do not necessarily represent those of their affiliated organizations, or those of the publisher, the editors and the reviewers. Any product that may be evaluated in this article, or claim that may be made by its manufacturer, is not guaranteed or endorsed by the publisher.
